# Crosstalk Between Oxidative Stress and Endoplasmic Reticulum (ER) Stress in Endothelial Dysfunction and Aberrant Angiogenesis Associated With Diabetes: A Focus on the Protective Roles of Heme Oxygenase (HO)-1

**DOI:** 10.3389/fphys.2019.00070

**Published:** 2019-02-11

**Authors:** Hatem Maamoun, Tarek Benameur, Gianfranco Pintus, Shankar Munusamy, Abdelali Agouni

**Affiliations:** ^1^Department of Medical Biochemistry and Molecular Biology, Faculty of Medicine, Ain Shams University, Cairo, Egypt; ^2^College of Medicine, King Faisal University, Al-Ahsa, Saudi Arabia; ^3^Department of Biomedical Sciences, College of Health Sciences, Qatar University, Doha, Qatar; ^4^Department of Pharmaceutical and Administrative Sciences, College of Pharmacy and Health Sciences, Drake University, Des Moines, IA, United States; ^5^Department of Pharmaceutical Sciences, College of Pharmacy, Qatar University, Doha, Qatar

**Keywords:** heme oxygenase-1 (Ho-1), endothelial dysfunction, angiogenesis, oxidative stress, ER stress, diabetes, hyperglycemia

## Abstract

Type-2 diabetes prevalence is continuing to rise worldwide due to physical inactivity and obesity epidemic. Diabetes and fluctuations of blood sugar are related to multiple micro- and macrovascular complications, that are attributed to oxidative stress, endoplasmic reticulum (ER) activation and inflammatory processes, which lead to endothelial dysfunction characterized, among other features, by reduced availability of nitric oxide (NO) and aberrant angiogenic capacity. Several enzymatic anti-oxidant and anti-inflammatory agents have been found to play protective roles against oxidative stress and its downstream signaling pathways. Of particular interest, heme oxygenase (HO) isoforms, specifically HO-1, have attracted much attention as major cytoprotective players in conditions associated with inflammation and oxidative stress. HO operates as a key rate-limiting enzyme in the process of degradation of the iron-containing molecule, heme, yielding the following byproducts: carbon monoxide (CO), iron, and biliverdin. Because HO-1 induction was linked to pro-oxidant states, it has been regarded as a marker of oxidative stress; however, accumulating evidence has established multiple cytoprotective roles of the enzyme in metabolic and cardiovascular disorders. The cytoprotective effects of HO-1 depend on several cellular mechanisms including the generation of bilirubin, an anti-oxidant molecule, from the degradation of heme; the induction of ferritin, a strong chelator of free iron; and the release of CO, that displays multiple anti-inflammatory and anti-apoptotic actions. The current review article describes the major molecular mechanisms contributing to endothelial dysfunction and altered angiogenesis in diabetes with a special focus on the interplay between oxidative stress and ER stress response. The review summarizes the key cytoprotective roles of HO-1 against hyperglycemia-induced endothelial dysfunction and aberrant angiogenesis and discusses the major underlying cellular mechanisms associated with its protective effects.

## Introduction

Endothelial dysfunction is considered as the structural alteration and functional impairment of vascular endothelial layer, characterized by a reduction in NO bioavailability and aberrant angiogenesis (Lerman and Burnett, 1992; Creager et al., 2003; Jamwal and Sharma, 2018). Endothelial dysfunction is a key player in the pathological onset of various cardiovascular complications associated with diabetes, whether affecting microvasculature including retinopathy, nephropathy, and neuropathy or macrovasculature such as ischemic heart disease and ischemic stroke (Ebrahimian et al., 2006). There is much scientific evidence showing that hyperglycemia, a characteristic manifestation of diabetes, is implicated in the development of endothelial dysfunction. Other molecular mechanisms involved in vascular endothelial perturbations that ensue in such a metabolic disorder include disruption of a large array of metabolic pathways within the endothelial cell leading to endoplasmic reticulum (ER) stress, oxidative stress, inflammation and apoptosis (Cimellaro et al., 2016; Incalza et al., 2018).

Heme oxygenase (HO) is a cytoprotective enzyme, which contributes to maintaining a healthy vascular endothelium. HO operates as a key rate-limiting enzyme in the process of degradation of the iron-containing molecule, heme, yielding the following byproducts: carbon monoxide (CO), iron, and biliverdin (Kikuchi et al., 2005; Ryter et al., 2006; Rochette et al., 2018). There are two main HO isoforms known (HO-1, HO-2), in addition to a putative third one (HO-3) (Nitti et al., 2017). Among the three isoforms, the role of HO-1 as a major protective enzyme is best documented. Its anti-oxidant, anti-apoptotic (Wang et al., 2010; Mishra and Ndisang, 2014; Tiwari and Ndisang, 2014), and anti-inflammatory (Hayashi et al., 1999; Lee and Chau, 2002; Morse et al., 2003; Ndisang and Jadhav, 2009) effects have attracted much interest in literature. The cytoprotective effects of HO-1 depend on multiple cellular processes including the generation of bilirubin, an anti-oxidant molecule, from the degradation of heme; the induction of ferritin, a strong chelator of free molecular iron; and the liberation of CO, responsible for HO-1 major anti-inflammatory and anti-apoptotic effects (Durante, 2003; Tiwari and Ndisang, 2014). However, the anti-inflammatory and anti-apoptotic effects of CO are only observed if CO is generated in low levels. Large amounts of CO can have lethal consequences because of the strong affinity of CO binding to the heme in hemoglobin and mitochondrial proteins. CO is a stable non-radical small gas molecule that is weakly soluble in water. It belongs to the family of gasotransmitters which emerged as important signaling molecules. CO signals inside the cell in several manners. For instance, CO stimulates guanylyl cyclase to form cGMP and regulates the action of some transcription factors (Mustafa et al., 2009; Garcia-Mata and Lamattina, 2013).

HO-1 is induced by a variety of pro-oxidant agents and stimuli, such as ultraviolet rays, heavy metals, inflammatory cytokines and iron-containing molecule, heme (Li et al., 2011). HO-1 is now recognized for playing anti-oxidative and cytoprotective roles both *in vitro* and *in vivo*. For example, mice deficient for HO-1 were found to spontaneously develop iron depots in kidneys and livers, tissue damage, chronic inflammation and oxidative structure modification of macromolecules (e.g., proteins, DNA) (Poss and Tonegawa, 1997a,b). Similar to animal models, the first reported human case of HO-1 deficiency involved significant iron tissue depots, growth deficiency, anemia and high susceptibility to reactive oxygen species (ROS)-mediated damage (Yachie et al., 1999). At the molecular level, it is believed that HO-1 protects vessel wall from pathological remodeling and endothelial cell dysfunction (Duckers et al., 2001; Durante, 2003; Awede et al., 2010; Hyvelin et al., 2010). Several methodologies were employed to induce HO-1 in the vessels with the use of pharmacological inducers being one of the most promising approaches (Awede et al., 2010; Hyvelin et al., 2010). Heme itself and its man-made analogs are strong pharmacological inducers of HO-1 and were found to protect against the development of cardiovascular diseases in several studies both *in vitro* and *in vivo* (Awede et al., 2010; Hyvelin et al., 2010; Li et al., 2011). A study conducted by Yang et al. (2015) where the serum of rats exposed to cigarette smoke was used to induce oxidative stress in human umbilical vein endothelial cells (HUVECs), has shown a significant decrease in endogenous production of ROS following the induction of HO-1 by hemin (Yang et al., 2015). Maamoun et al. (2017) found that the pharmacological induction of HO-1 using Cobalt-protoporphyrin (CoPP) reduced ROS production in HUVECs exposed to intermittent high glucose.

With regards to the anti-inflammatory effects of HO-1, Chang et al. (2014) have shown that the treatment of HUVECs with iodine contrast medium caused anti-proliferative and inflammatory reactions, and enhanced the expression of intercellular adhesion molecule (ICAM)-1 and adhesion molecules receptors while cells co-incubated with the HO-1 inducer were completely protected (Chang et al., 2014). The cytoprotective role of HO-1 has also been illustrated in cancer cells, where one study has demonstrated that the upregulation of HO-1 in renal cancer cells promoted their survival capacity via the induction of the expression of pro-survival molecule Bcl-xL and decreased expression of Beclin-1 and LC3B-II, that are involved in the process of autophagy, an effect that has been reversed by HO-1 knockdown (Banerjee et al., 2012). Furthermore, in vascular cells, it has been found that HO-1 induction protected HUVECs from high glucose mediated cell death through the reduction of caspases 3 and 7 activation (Maamoun et al., 2017).

In the current article, we have reviewed the major mechanisms contributing to endothelial dysfunction, the key initial step in the onset of atherosclerotic process, in the context of diabetes and hyperglycemia. Furthermore, we have reviewed the cytoprotective roles of HO-1 against diabetes- and hyperglycemia-induced endothelial dysfunction and aberrant angiogenesis and discussed the major underlying molecular mechanisms associated with these protective effects with special emphasis on signaling pathways related to oxidative stress and ER stress response.

## Endothelial Dysfunction and Hyperglycemia: Key Molecular Disturbances

The endothelium is a single cell layer that forms the interface between blood stream and adjacent tissues. Over the recent decades the complexity of this selectively permeable barrier and its important contribution to controlling vascular homeostasis have been established (Michiels, 2003; Khazaei et al., 2008; Jamwal and Sharma, 2018). The endothelium allows the selective passage of certain substances such as nutrients through the vessel wall to the adjacent tissues. The endothelium is recognized as an endocrine organ that is able to produce and secrete many hormones and mediators which are crucial for the optimal functioning of the vasculature such as factors regulating vascular tone, coagulation, immune response and growth of adjacent vascular cells (Khazaei et al., 2008; Jamwal and Sharma, 2018). In normal physiological conditions, vascular endothelial cells are exposed to plasma blood sugar levels between 3.8 and 6.1 mmol/L. Exposure of endothelial cells to glucose levels of 11.1 mmol/L and above is regarded as a diabetic condition [the national institute for health and care excellence (NICE) guidelines, 2015]. However, unlike *in vivo* conditions, in cell culture, the definition of high glucose varies considerably according to the cell model used, depending on glucose levels in culture medium where the cells are being selected and harbored. Two examples that demonstrate such variability are the endothelial cell line EA.hy926 and HUVECs, where the former is propagated in culture medium containing 25 mM glucose, whereas the latter is routinely grown in a culture medium containing 5.5 mM of glucose (Maamoun et al., 2017). As a result, 25 mM glucose would be considered to be a high concentration of glucose for HUVECs, but is a standard level in EA.hy626 endothelial cell culture medium. Alterations occur in both the micro- and macrovasculature in response to hyperglycemia. An abnormally high glucose concentration can disrupt the balance within the endothelial cell and a state of ‘endothelial cell dysfunction’ results. Endothelial cell dysfunction is defined by the presence of one or more of the following characteristics: (I) low bioavailability of NO, (II) impaired endothelium-dependent relaxation, (III) weak fibrinolytic capability, (IV) excess of production of growth factors, adhesion molecules and pro-inflammatory molecules (e.g., cytokines), oxidative stress, and aberrant angiogenesis (Cade, 2008).

### Hyperglycemia-Mediated Oxidative Stress and Endothelial Injury

**FIGURE 1:**
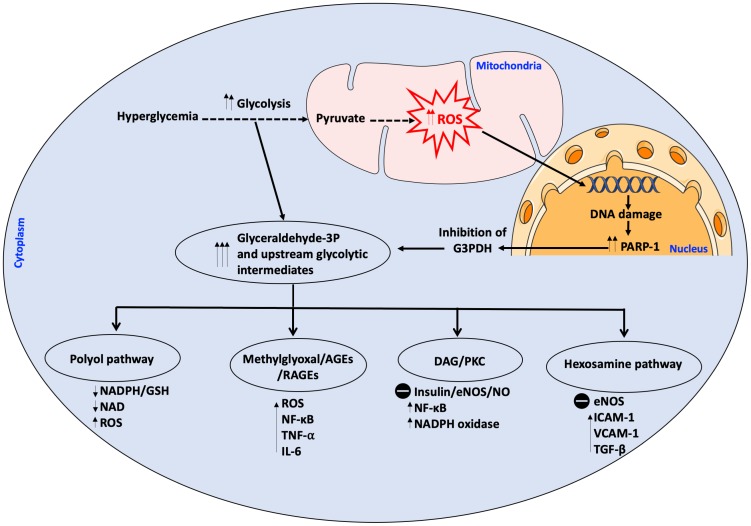
Intracellular hyperglycemia results in increased production of mitochondrial ROS. ROS cause DNA damage releasing PARP-1 which inhibits G3PDH. This results in the accumulation of upstream glycolytic intermediates. Other pathways are activated as a result of the accumulation of glycolytic intermediates upstream of G3P which have detrimental effects on the cell; these pathways are; (1) Polyol pathway which depletes the cell of its antioxidant GSH system due to scavenging of NADPH, thereby augmenting ROS production, (2) Methylglyoxal which is a precursor to AGEs/RAGEs which activate pro-inflammatory and pro-oxidant molecules, (3) DAG/PKC which impairs insulin signaling and eNOS activity, activates pro-inflammatory and pro-oxidant molecules, (4) Hexosamine pathway with the resultant activation of pro-inflammatory molecules, inhibition of eNOS at the Akt activation site and activation of TGF-β with increase in ROS. All these pathways increase ROS in a positive feedback loop. AGEs, advanced glycation end products; DAG, diacylglycerol; eNOS, endothelial NO synthase; G3PDH, Glyceraldehyde 3-phosphate dehydrogenase; GSH, Glutathione; ICAM-1, intracellular adhesion molecule-I; IL-6, interleukin 6; NAD, nicotinamide adenine dinucleotide; NADP, NAD phosphate; NF-κB, nuclear factor kappa B; NO, nitric oxide; PARP-1, poly ADP ribose polymerase 1; PKC, protein kinase C; RAGE, AGEs receptor; ROS, reactive oxygen species; TGF-β, transforming growth factor β; TNF-α, tumor necrosis factor α; VCAM-1, vascular cell adhesion molecule 1. The symbol 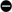 means inhibition.

Macrovascular and microvascular complications have been shown to be mainly or partly dependent on hyperglycemia (Zoungas et al., 2012). Hyperglycemia can induce vascular endothelial damage through different pathways: (I) enhanced polyol activity, causing sorbitol and fructose accumulation; (II) enhanced production of advanced glycation end products (AGEs); (III) activation of mitogenic protein kinase C (PKC); (IV) heightened hexosamine flux pathway and (V) a decrease of body anti-oxidant defenses (Brownlee, 2001; Kornfeld et al., 2015). There is considerable evidence that these biochemical pathways can be induced by excessive generation of ROS, leading to increased oxidative stress in a positive feedback loop (Son, 2012). Oxidative stress occurs when the concentrations of ROS exceed those of anti-oxidant neutralizing species, such as glutathione (GSH) and HO. ROS are a heterogeneous population of molecules including free radicals, such as hydroxyl radical (OH^-^), superoxide anion (O_2_^-^), peroxyl (RO_2_), and hydroperoxyl (HRO_2_^-^), and non-charged species, such as hydrogen peroxide (H_2_O_2_) and hydrochloric acid (HCl) (Pieper et al., 1997). Under intracellular hyperglycemic conditions, excessive production of ROS develops through several mechanisms, the most important of which is excessive activation of mitochondrial electron transport chain. Other sources of ROS include glucose-induced activation of NADPH oxidase (NOX) and xanthine oxidase (Nishikawa et al., 2000). This excessive ROS production inflicts DNA damage resulting in poly ADP ribose polymerase (PARP)-1 activation, which in turn inhibits the glycolytic enzyme glyceraldehyde-3-phosphate dehydrogenase (G3PDH) resulting in the pile-up of upstream glycolytic metabolites, with the resulting influx into polyol pathway, hexosamine pathway, diacylglycerol (DAG) and PKC pathway, as well as generation of AGEs (Du et al., 2003) (Figure 1).

**FIGURE 2:**
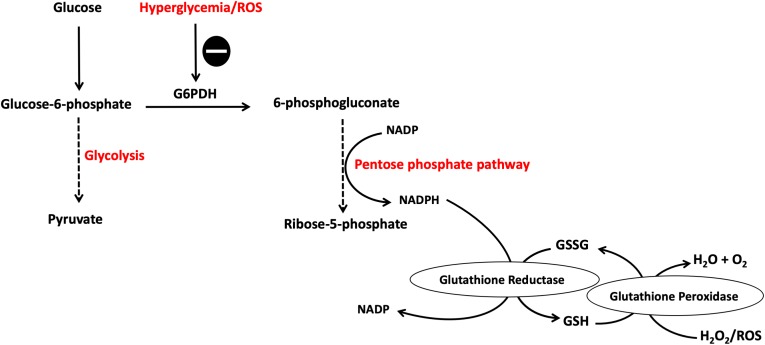
Pentose phosphate pathway (PPP) is inhibited by hyperglycemia and ROS. Hyperglycemia-induced ROS cause the inhibition of G6PDH which is the rate limiting enzyme for this pathway. This results in reduced NADPH production, a cofactor fundamental in the GSH/peroxidase antioxidant system, with the consequent buildup of more ROS causing a vicious circle of oxidative stress induction. G6PDH, glucose-6-phosphate dehydrogenase; GSH, glutathione; NADPH, reduced NAD phosphate. The symbol 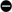 means inhibition.

**FIGURE 3:**
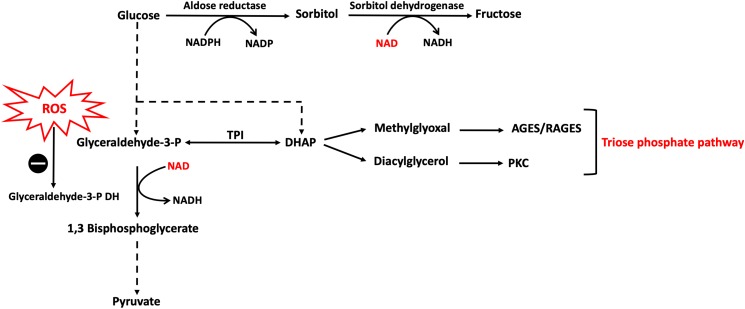
Polyol and triose phosphate pathways activation caused by hyperglycemia-induced ROS. Hyperglycemia results in an excess of ROS production which inhibits G3PDH with the consequent accumulation of glycolytic intermediates upstream to G3P leading to increase flux into other pathways, namely polyol and triose phosphate. In polyol pathway, NAD reducing equivalent is consumed by sorbitol dehydrogenase reaction leading to reductions in NAD levels inside the cell which further confounds the inhibition of G3PDH. DHAP, dihydroxyacetone phosphate; G3PDH, glyceraldehyde 3-phosphate dehydrogenase; NAD, nicotinamide adenine dinucleotide; TPI, triose phosphate isomerase. The symbo 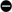 means inhibition.

Glucose, through the polyol pathway, is converted into polyalcohol sorbitol by aldose reductase utilizing NADPH as a cofactor, which in turn reduces intracellular concentrations of NADPH resulting in the inhibition of glutathione/peroxidase anti-oxidant system, which is dependent on NADPH as a cofactor, and consequently induces overproduction of H_2_O_2_ and ROS in general. Moreover, ROS accumulation inhibits glucose-6-phosphate dehydrogenase (G6PDH), which is the rate limiting enzyme for pentose shunt pathway that is fundamental to the maintenance of reducing equivalents for the glutathione/peroxidase system anti-oxidant system, amplifying the oxidative stress (Figure 2). Furthermore, sorbitol is oxidized to fructose by sorbitol dehydrogenase using NAD as a co-factor, increasing the intracellular ratio of NADH/NAD^+^, exacerbating the inhibition of G3PDH which is dependent on NAD as a cofactor; thus amplifying the accumulation of glyceraldehyde-3-phosphate (G3P) with the consequent flux into triose phosphate pathway via triose phosphate isomerase (TPI) yielding dihydroxyacetone phosphate (DHAP). Higher levels of triose phosphate promote the formation methylglyoxal and DAG. Methylglyoxal is a precursor of AGEs, which are formed by binding of methylglyoxal to the free amino groups of intracellular and extracellular proteins (Allaman et al., 2015; Maessen et al., 2015) (Figure 3).

DAG activates certain PKC isoforms by binding to their membrane-bound receptors. Consequently, active PKC isoforms may contribute to hyperglycemia-induced vascular endothelial damage by inhibiting insulin-stimulated endothelial NO synthase (eNOS) expression and NO production in endothelial cells, increasing thus the activity of nuclear factor kappa B (NF-κB) and the pro-oxidant enzyme NOX (Giri et al., 2018). Under hyperglycemic conditions, PKC-α and PKC-γ, were reported to be able to enhance NOX activity (Venugopal et al., 2002; Dasu et al., 2008). PKC-β, a molecular mediator of hypertrophic responses, was also found to be selectively over-activated in vascular and cardiac cells of animal models of diabetes. Liu et al. (2012) observed that the selective inhibition of PKC-β, improved left ventricular function and structure in a diabetes animal model of streptozotocin-injected rats. More recently, it was reported that the activation of PKC-α in the intestine of streptozotocin-induced diabetic mice contributed to the enhanced uptake of iron leading to iron loading that contributes to diabetic complications (Zhao et al., 2018). Furthermore, the inhibition of PKC-ζ was shown recently to improve insulin sensitivity and uptake of glucose in rat adipocytes rendered insulin-resistant by incubating them in high glucose and high insulin concentrations (Lu et al., 2018).

Hyperglycemia-induced ROS overproduction is also caused by the interaction of AGEs with their receptors (RAGEs) (Rochette et al., 2014). This interaction generates intracellular ROS and activates NF-κB, which modulates the expression of a many genes associated with inflammation and vascular remodeling, including interleukin (IL)-6, tumor necrosis factor (TNF)-α, ICAM-1, vascular cell adhesion molecule (VCAM)-1 and monocyte chemotactic protein-1 (MCP-1) (Bierhaus et al., 2001; Younce et al., 2010). Furthermore, AGEs which are present in the extracellular matrix can decrease the availability of NO, reducing thereby endothelium-dependent vasodilation (Xu B. et al., 2005). Finally, the activation of the hexosamine pathway contributes to the vascular damage induced by hyperglycemia owing to its capacity to disturb multiple cellular processes, including signal transduction, gene transcription, cell survival, and proteasome-mediated degradation (Love and Hanover, 2005). The activation of the hexosamine pathway can inhibit eNOS activity by impairing insulin receptor activation and signal transduction and increasing ROS production (Rajapakse et al., 2009a,b).

### ER Stress and Endothelial Dysfunction in Diabetes

In addition to hyperglycemia-induced oxidative stress, ER stress was also linked to various features of endothelial dysfunction in diabetics with decreased NO bioavailability and aberrant angiogenesis being two prominent features (Sheikh-Ali et al., 2010). Aberrant angiogenesis may be either an excessive angiogenic response (e.g., retinopathy, nephropathy) or a deficiency in angiogenesis (e.g., disturbed wound healing, reduced sprouting of collateral vessels, neuropathy, ischemia and peripheral vascular disease) (Kota et al., 2012). Under normal physiological conditions, the ER plays a central role in protein synthesis and co-translational protein modification through the folding of newly synthesized proteins by native disulfide bond formation to attain the final stable conformational state prior to being directed to their final destination whether it is to be secretory or membrane bound proteins. The homeostasis of ER is put at stress when the influx of the newly synthesized misfolded or unfolded polypeptide chains exceeds the repair and refolding capacity of the ER; this condition is commonly referred to as ER stress response (Walter and Ron, 2011).

**FIGURE 4:**
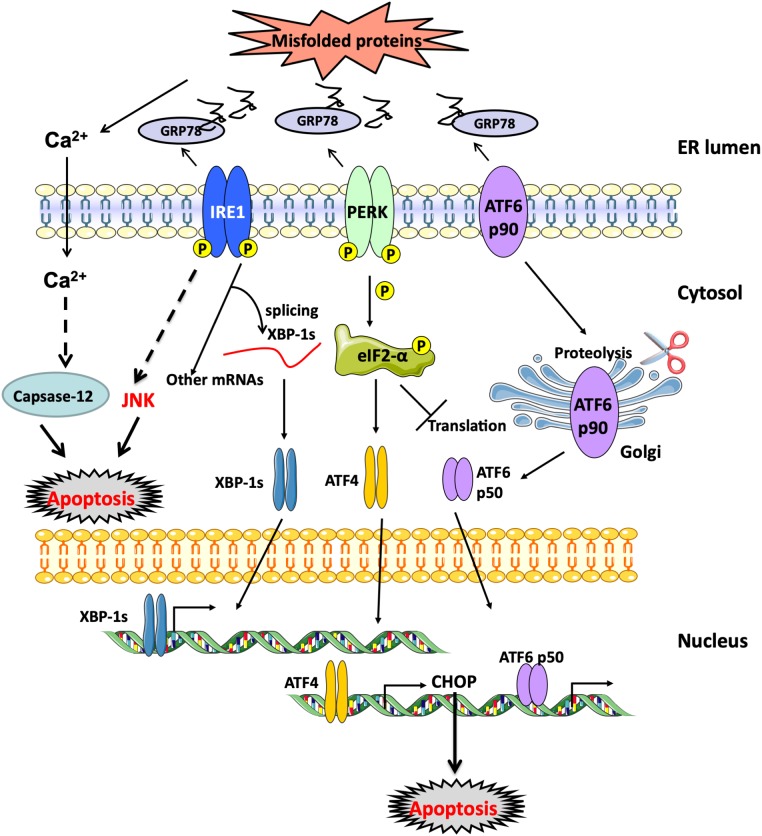
UPR response activation. ER membrane-bound proteins, PERK, ATF-6 and IRE-1α, are activated when unfolded proteins accumulate within the ER lumen and GRP78/BiP dissociates from them. Downstream effects of PERK involve the phosphorylation of eIF-2α resulting in the attenuation of protein translation with the preferential increase in ATF-4 expression. ATF-6 (p90) translocates to the Golgi body where it undergoes proteolytic cleavage. Active cleaved ATF-6 (p50) translocates then to the nucleus and stimulates the expression of molecular chaperones to aid in the folding process. Finally, active IRE-1α through its endoribonuclease activity undergoes intron splicing of *XBP-1* mRNA to synthesize the active spliced XBP-1s which binds to the promoter regions of several genes involved in the synthesis of chemical chaperones and ERAD proteins to restore normal protein homeostasis as part of the UPR rescue response. ATF-6, activating transcription factor 6; BiP, binding immunoglobulin protein; eIF-2α, Eukaryotic translation initiation factor 2α; ERAD, endoplasmic-reticulum-associated protein degradation; GRP78, 78-kDa glucose regulated protein; IRE-1α, inositol-requiring enzyme 1α; PERK, PKR-like ER kinase; XBP-1, X-box binding protein 1.

Endoplasmic reticulum stress response develops both in type-1 or type-2 diabetes mellitus due to the presence of hyperglycemia that increases the demand for the synthesis of enzymatic machinery necessary to carry out complete glucose oxidation through glycolysis, tricarboxylic acid cycle (TCA) and oxidative phosphorylation by mitochondrial electron transport chain (Flamment et al., 2012; Hu et al., 2017). This increase in protein load on the ER increases the chances of accumulation of unfolded or poorly folded proteins inside the ER by the formation of incorrect or non-native disulfide bonds due to mispairing of cysteine residues in the protein being synthesized (Lisa et al., 2012). When this occurs, the cell triggers the unfolded protein response (UPR), which aims to resolve the problem by three different mechanisms working synergistically together: (I) Enhancement of gene expression of molecular chaperones to support and improve the correct folding of proteins; (II) Activation of ER-associated degradation machinery (ERAD) to get rid of aberrant proteins; and (III) Attenuation of translation rate to slow down the entry of new proteins to the ER lumen (Flamment et al., 2012). The molecular system involved in such response includes three membrane-bound proteins that orchestrate the whole process, namely inositol requiring enzyme (IRE)-1α, protein kinase RNA-like ER kinase (PERK) and activating transcription factor (ATF)-6, which under basal conditions are kept in an inactive state by binding to 78 kDa glucose-regulated protein (GRP)-78 or binding immunoglobulin protein (BiP); however, when there is an increase of misfolded proteins, GRP78/BiP preferentially binds to them hence relieving those ER effectors from their inhibition state. The dissociation of GRP78 from the three effectors is followed by the dimerization and hence activation of both IRE-1α and PERK, and the transfer of ATF-6 to the Golgi body for proteolytic processing activation before the final active ATF-6 translocates to the nucleus and stimulates the transcription of target genes. The UPR is a physiological pro-survival process which attempts to restore normal homeostatic state of the ER within the cell; however, if the stress condition remains unresolved, these three effectors involved in the UPR can also activate multiple inflammatory responses and eventually may lead to cell death through the activation of several pro-apoptotic sub-pathways (Gardner and Walter, 2011; Walter and Ron, 2011; Hassler et al., 2012; Gardner et al., 2013) (Figure 4).

Previous studies have demonstrated the role of ER stress response in endothelial cell inflammation and cell death in vascular diseases associated with diabetes. Work by Chen et al. (2012) demonstrated that the ATF-4-mediated activation of signal transducer and activator of transcription 3 (STAT3) is responsible for inflammation, vascular endothelial damage and loss of angiogenic capacity in streptozotocin-induced wild-type mouse model of type-1 diabetes, while ATF-4 knockout mice were protected. Other *in vitro* studies have also shown that the blockade of STAT3 reduced high glucose-mediated ER stress response activation, suggesting a crosstalk between ER stress and inflammatory signaling pathways. Moreover, another study of diabetic cardiovascular complications in streptozotocin-induced mouse model of diabetes found that the activation of the epidermal growth factor receptor (EGFR) tyrosine kinase signaling pathway participated in the induction of ER stress response and microvascular dysfunction in these mice. Enhanced EGFR phosphorylation was associated with the activation of the PERK/eukaryotic initiation factor (eIF)-2α/ATF-4 axis. The pharmacologic blockade of the kinase activity of EGFR ameliorated endothelium-dependent relaxation and increased NO production (Galan et al., 2012). Previously, Agouni et al. (2011) investigated in a mouse model of diet-induced obesity the impact of whole-body alleviation of ER stress response through the specific liver-deletion of protein tyrosine phosphatase (PTP)-1B, a protein located on the luminal surface of the ER that was shown to be implicated in the activation of ER stress (Owen et al., 2013), on endothelial cell function. Authors reported that the alleviation of ER stress response improved endothelium-dependent vasodilation in mouse aortas and improved vascular NO bioavailability (Agouni et al., 2014).

The role of ER stress in ischemia-induced neovascularization was demonstrated in type-2 diabetes genetic mouse model (*db/db*). The chemical chaperones, tauroursodeoxycholic acid (TUDCA) and 4-phenylbutyric acid (4-PBA), known to alleviate ER stress, improved hind-limb ischemia after femoral artery ligation. These small molecules reduced the expression of ATF-4 and C/EBP homologous protein (CHOP), enhanced blood flow recovery following ligation, and increased the expression of pro-angiogenic factors (Amin et al., 2012). There is also growing evidence that ER stress response might play an important role in promoting angiogenesis, and therefore may act as a novel mediator of the angiogenic process. This evidence implicates these pathways in the upregulation of angiogenic factors such as vascular endothelial growth factor (VEGF)-A, fibroblast growth factor (FGF), IL-8, and endothelin (ET)-1 (Paridaens et al., 2014). Notably, it was shown that ER stress-mediated overexpression of VEGF-A was dependent on the IRE-1α/X-box binding protein (XBP)-1, PERK/ATF-4, as well as ATF-6 arms of ER stress response. The upregulation of VEGF-A expression by UPR effectors was also reported in diabetic nephropathies and retinopathies, indicating the involvement of UPR and ER stress responses in the enhancement of angiogenesis beyond the physiological conditions (Ghosh et al., 2010). However, Maamoun et al. (2017) have reported more recently that the chemical chaperone, 4-PBA prevented intermittent high glucose-induced impaired angiogenic capacity in HUVECs characterized by a weaker ability to form tube-like structures on a matrigel matrix and a reduced the expression of VEGF-A. Nonetheless, the exact molecular mechanisms involved in the switch between pro- and anti-angiogenic stimulation by ER stress response are not fully understood.

Endoplasmic reticulum stress also plays a key role in the onset of atherosclerosis in diabetes, a major consequence of endothelial dysfunction. Several independent risk factors for cardiovascular diseases, including hyperglycemia (Werstuck et al., 2006), hyper-homocysteinemia (Werstuck et al., 2001), obesity (Ozcan et al., 2004; Agouni et al., 2011), and dyslipidemia have been associated with ER stress, indicating that it may be a converging molecular link for atherogenesis (Cunha et al., 2008). The activation of UPR response has been observed at all stages of atherogenesis (Zhou et al., 2005). ER stress inducers can promote lipid tissue accumulation by activating the sterol regulatory element binding proteins (SREBP), which control the transcriptional regulation of lipid synthesis and transport in endothelial cells (Colgan et al., 2007). ER stress inducers also activate NF-κB, which promotes the expression of pro-inflammatory and pro-coagulant genes contributing thus to atherogenesis (Jiang et al., 2003). Moreover, ER stress effector IRE-1α causes the activation of c-Jun N-terminal kinases (JNK) which impairs insulin signaling reducing thus insulin-stimulated eNOS activity with subsequent reduction of NO production (Andreozzi et al., 2007). ER stress has also been shown *in vivo* and *in vitro* to activate pro-apoptotic caspases and cell death of human endothelial cells (Xu C. et al., 2005; Maamoun et al., 2017).

There is also evidence suggesting a crosstalk between ER stress and oxidative stress. Maintaining ER balance is intimately associated with oxidative state of the cell. In contrast to cytosol, the ER lumen is an oxidizing environment characterized by an elevated ratio of oxidized to reduced glutathione (GSSG/GSH) which encourages the proper native disulfide bond formation (van der Vlies et al., 2003). During disulfide bond-dependent protein folding, proteins are oxidized to form disulfide bonds by protein disulfide isomerase which allows proper polypeptide rearrangement to reach their final native conformational state. Any mispairing of cysteine residues would result in the formation of non-native disulfide bonds that prevent proteins from achieving their native optimal conformation, a condition that results when there is an abnormally high request for protein synthesis as in the case of hyperglycemia. In addition, hyperglycemia-induced AGEs accumulation as well as hexosamine and polyol pathways activation can impair the correct protein folding, leading to the activation of UPR and ER stress responses (Chakravarthi and Bulleid, 2004).

**FIGURE 5:**
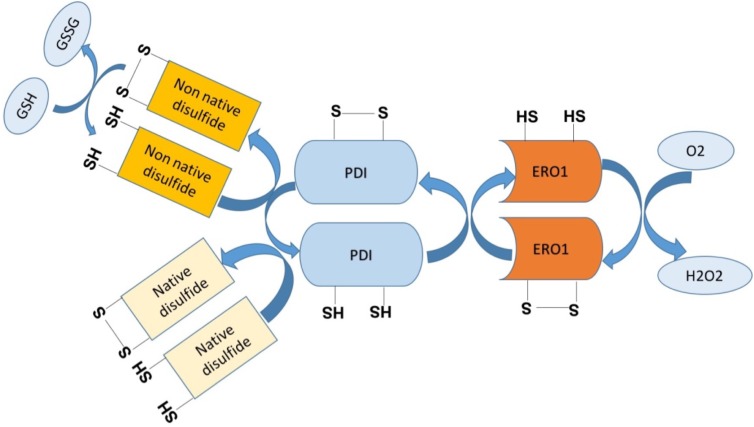
Schematic representation showing ER protein folding as a source of ROS. During ER stress response, there is accumulation of misfolded proteins within the ER where non-native disulfide bonds form resulting in the depletion of glutathione (GSH) that is trying to correct the aberrant bonds. IRE-1α, inositol-requiring enzyme 1 α; PDI, protein disulfide isomerase.

**FIGURE 6:**
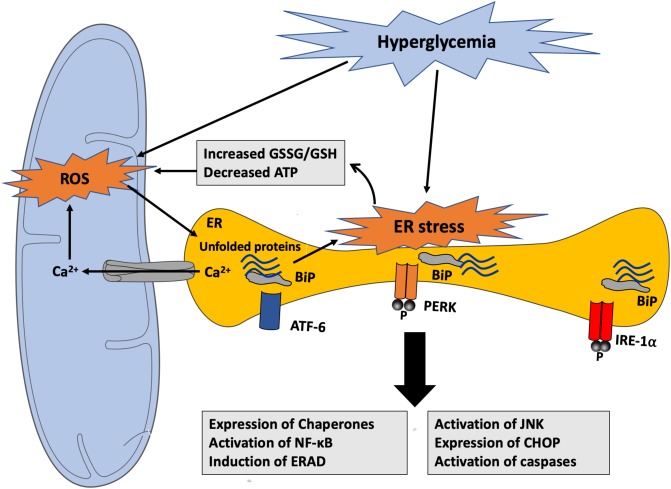
Hyperglycemia-induced ROS generation and activation of ER stress response. Hyperglycemia-generated ROS can promote the accumulation of unfolded proteins within the ER contributing to ER stress response through different mechanisms. ER dysfunction causes GSH depletion, Ca^2+^ leak from the ER lumen into the cytosol and into the mitochondria, and ATP depletion that can impair its structural and functional integrity and stimulate mitochondrial ROS production. ROS, in turn, can alter protein folding, thereby inducing unfolded protein accumulation within the ER lumen. ATF-6, activating transcription factor 6; BiP, binding immunoglobulin protein; CHOP, C/EBP homologous protein; ERAD, endoplasmic-reticulum-associated protein degradation; GSH, Glutathione; GSSG, oxidized Glutathione; IRE-1α, inositol-requiring enzyme 1α; JNK, c-Jun N-terminal kinases; NF-κB, nuclear factor kappa B; PERK, PKR-like ER kinase; ROS, reactive oxygen species.

In normal conditions, during protein folding, electrons are transferred from targeted cysteine residues in the polypeptide chain being synthesized forming native disulfide bonds to bring the protein structure to its final conformational state. This reaction is catalyzed by two crucial enzymes, namely protein disulfide isomerase (PDI) and ER oxidoreductase (ERO)-1α, which aid in this electron transfer from the target cysteine residues to molecular oxygen, generating H_2_O_2_; however, in the presence of a high protein folding load, as in hyperglycemia, there is an increase in non-native disulfide bond formation, which results in GSH consumption as a protective mechanism. This results in GSH depletion contributing thus to excessive ROS generation and consequent development of oxidative stress (van der Vlies et al., 2003) (Figure 5). Moreover, hyperglycemia-induced oxidative stress is suggested to contribute to the inactivation of disulfide isomerases resulting in the buildup of unfolded proteins in addition to the misfolded ones (Tu and Weissman, 2004). Since protein folding is a highly energy-dependent process, protein misfolding-induced ATP depletion leads to more glucose consumption to promote mitochondrial oxidative phosphorylation to increase ATP synthesis and consequently increasing ROS production to pathological levels. As a result of unfolded/misfolded protein accumulation, Ca^2+^ leak ensues from the ER lumen into the cytosol and increased intracellular Ca^2+^ levels further stimulate mitochondrial ROS production. In fact, Ca^2+^ increases the permeability of inner mitochondrial membrane and blocks the electron transport chain at the level of complex III, thereby causing electron leak to molecular O_2_, thus exacerbating the ongoing ROS production (Gorlach et al., 2006) (Figure 6). Taken together, there is substantial evidence that both ER stress and oxidative stress are concomitantly induced in hyperglycemic conditions and they play an important role in the perturbations that ensue in the vascular endothelium leading to the development of endothelial dysfunction in diabetes.

### Mitochondrial Dysfunction and Endothelial Dysfunction in Diabetes

**FIGURE 7:**
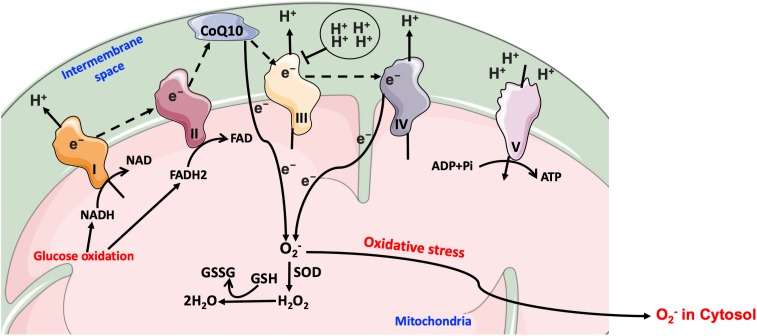
Mitochondrial electron transport chain in hyperglycemia. Under hyperglycemic conditions, higher levels of glucose-derived pyruvate increase the flux of NADH and FADH_2_ into the electron transport chain and consequently increase the proton gradient across the mitochondrial membrane. The transfer of free electrons to complex III is blocked, leading the electrons to be donated to coenzyme Q10, which then transfers electrons to molecular oxygen, thereby generating O_2_^-^. CoQ10, coenzyme Q10; e^-^, electrons; O_2_^-^, superoxide; GSH, glutathione; GSSG, oxidized glutathione; H_2_O_2_, hydrogen peroxide; Pi, inorganic phosphate; SOD, O_2_^-^ dismutase.

Much evidence suggests that mitochondrial dysfunction is a major source for hyperglycemia-mediated generation of ROS through the electron transport chain, which contributes to oxidative damage pathways as discussed above, namely the polyol, triose phosphate, hexosamine, and AGEs pathways (Nishikawa et al., 2000). Normally, NADH and FADH_2_ released from the TCA cycle translocate from the mitochondrial matrix to the inner mitochondrial membrane, where they will donate free electrons to the series of mitochondrial membrane-bound complexes. Free electrons are transferred through mitochondrial complexes I, II, III and cytochrome c (IV), until they are finally transferred to molecular oxygen to form water. The electron transfer across these complexes generates a gradient of protons in the mitochondrial intermembrane space, generating thus a proton potential gradient across the inner mitochondrial membrane, which activates ATP synthesis. When electrons are moving from complex III to complex IV, ROS radicals are generated in small amounts during normal oxidative phosphorylation process; these ROS radicals are then captured by enzymatic anti-oxidants such as glutathione (GSH) and superoxide dismutase (SOD) isoforms. Under hyperglycemic conditions, higher levels of glucose-derived pyruvate and acetyl-CoA enter the TCA cycle increasing thus the generation of NADH and FADH_2_ that, in turn, transfer free electrons into the electron transport chain and consequently massively increase proton gradient through the mitochondrial membrane. This abnormally high proton gradient blocks complex III, forcing free electrons to move back to coenzyme Q10, which then transfers these electrons to molecular oxygen in a process known as electron leak, thereby generating O_2_^-^ anions (Rolo and Palmeira, 2006). As mentioned above, in normal physiological conditions there is still some mitochondrial ROS being generated during the electron migration at complex IV; however, it is within the capacity of the glutathione/peroxidase system and SOD to scavenge it and prevent buildup of oxidative stress (Figure 7).

### NADPH Oxidase (NOX) Activation and Endothelial Dysfunction in Diabetes

In addition to the mitochondrial dysfunction being a major source of hyperglycemia-induced ROS overproduction, studies have shown there are other principle sources that include: NOX and uncoupled eNOS (Inoguchi et al., 2000; Volpe et al., 2018). The NOX family of enzymes were identified first in phagocytic cells, neutrophils and macrophages. They are the only family of enzymes with a primary function to generate ROS (H_2_O_2_, O_2_^-^) by catalyzing the electron transfer from cytosolic NADPH to molecular O_2_ across plasma and lysosomal membranes (Thannickal and Fanburg, 2000; Salazar, 2018). There is a total of seven isoforms identified so far which are abundantly expressed in phagocytic cells. These isoforms include, NOX1, NOX2, NOX3, NOX4, NOX5, DUOX1 and DUOX2. Four of these are expressed by endothelial cells, NOX1, NOX2, NOX4, and NOX5 (Salazar, 2018). In endothelial cells, NOXs are believed to produce moderate levels of ROS which are needed for redox signaling during normal cell metabolism process. However, in certain pathological states, NOX expression and activity in the vasculature was found to be increased, thus contributing to excess release of ROS.

NOX isoforms consist of a catalytic subunit “NOX,” which catalyzes the transfer of free electrons from cytosolic NADPH to molecular oxygen. Specifically, for NOX-1, 2, and 4, additional regulatory subunits are also required for a complete activation of the complex. These subunits include p22^phox^, p47^phox^, p67^phox^, and p40^phox^. By contrast to the other isoforms, NOX-5 is a unique polypeptide subunit which is regulated by Ca^2+^. One additional difference between these isoforms is that while NOX-1, 2, and 5 generate superoxide O_2_^-^, NOX-4 generates the non-charged radical H_2_O_2_ (Drummond and Sobey, 2014). The subunit p47^phox^ is pivotal for NOX activation and is frequently named the ‘organizer subunit’ since it is crucial for the formation of an active NOX complex. It resides within the cytosol in resting state; however, upon its phosphorylation at several serine residues, it undergoes conformational changes to expose its intramolecular SH-3 domain which interacts with the cytosolic activator p67^phox^ subunit, and chaperones it to the membrane-bound subunits, NOX and p22^phox^, to finally form the active NOX complex (Schulz et al., 2011; Drummond and Sobey, 2014).

Hyperglycemia, free fatty acids, and oxidized low-density lipoprotein (LDL) have been shown to enhance endothelial NOX activity (Shen, 2010). Vessels isolated from diabetic subjects exhibit increased O_2_^-^ production and higher expression levels of several NOX subunits (p22^phox^, p47^phox^, and p67^phox^), suggesting that NOX isoforms are more active in diabetes (Pitocco et al., 2010). The excess of O_2_^-^ can rapidly react with NO to form ONOO^-^ which, in turn, oxidizes tetrahydrobiopterin (BH_4_), thus making it unavailable for interaction with eNOS. In the presence of low amounts of BH_4_, eNOS becomes uncoupled and donates free electrons to O_2_ instead of L-arginine, producing therefore O_2_^-^ instead of NO. Clinical studies demonstrated that BH_4_ supplementation improved endothelium-dependent vasodilation in diabetic patients, highlighting the important role of uncoupled eNOS in endothelial cell dysfunction in diabetes (Son, 2012). Collectively, hyperglycemia is tightly associated with NOX activation, mitochondrial dysfunction, and ER stress response. These cell responses are unequivocally major mechanisms underpinning ROS overproduction and oxidative stress-mediated endothelial cell damage in diabetes.

## Endothelial Dysfunction and Altered Angiogenic Capacity in Diabetes: a Focal Role for NO and ROS

Blunted eNOS activity and/or protein expression and subsequent decreased NO bioavailability is a hallmark of endothelial dysfunction associated with diabetes (Hirata et al., 2010). Protein expression of eNOS is tightly controlled by several molecular processes, which include transcriptional and post-transcriptional regulation of mRNA expression (Searles, 2006), and post-translational regulation of the protein structure and function where the role of Akt activity is crucial in enhancing the phosphorylation of eNOS at Ser1177 causing its activation (Shen et al., 2006). In early stages of hyperglycemia-induced cellular stress, high glucose-induced ROS production alters Ca^2+^ homeostasis leading to Ca^2+^ leaking from ER stores into the cytosol (Ding and Triggle, 2010; Triggle and Ding, 2010). As a result, intra-ER Ca^2+^ levels fall below a critical level interfering thus with the activity of many ER chaperones which depend on Ca^2+^ availability inside the ER lumen and hence interfere with proper protein folding leading to activation of UPR and eventually apoptosis (Morgan et al., 2008). Furthermore, the resulting increase in cytosolic Ca^2+^ induces the activation of eNOS which can produce high amounts of NO that can then quickly react with O_2_^-^ to form ONOO^-^, thereby oxidizing BH_4_ and eventually leading to eNOS uncoupling and a reduction in the bioavailability of NO (Ding and Triggle, 2010). In advanced stages, when ER stress reaches a point where it cannot be resolved, the cell must activate pro-apoptotic signals. Endothelial cells incubated in high glucose, activate IRE-1α -mediated cell death response following a disruption Ca^2+^ homeostasis due to an increase in the levels of ROS (Bishara and Ding, 2010).

The activation IRE-1α stimulates JNK and p38 mitogen-activated protein kinases (MAPK) pathways triggering thus the downstream pro-apoptotic mediators, apoptosis signal-regulating kinase (ASK)-1 and caspase-12, via CHOP activation (Xu C. et al., 2005). ASK-1 can cause NO deficiency by inhibiting eNOS through reduced phosphorylation at Ser1177 site (Yamashita et al., 2007). A study conducted by Galan et al. (2014) demonstrated in coronary artery endothelial cells that were subjected to high glucose or ER stress inducer, tunicamycin, the role of NOX in the suppression of eNOS promotor region and decreased phosphorylation. These effects were restored by a chemical chaperone, 4-PBA, that helped resolve ER stress. They also reproduced similar results *in vivo* by showing a significant reduction in endothelium-dependent and independent relaxation in control mice compared with p47^phox^-deficient mice, indicating the involvement of p47^phox^ subunit of NOX complex as a molecular link for ER stress-induced reduction in NO production and the consequent vascular endothelial dysfunction (Galan et al., 2014). More recently, it has been reported that high glucose-induced ER stress caused an increase in the phosphorylation of p47^phox^ at Ser345 (Maamoun et al., 2017). This critical phosphorylation was found to precede the activation of NOX (Perisic et al., 2004). Nevertheless, regardless of the exact mechanisms involved in the inhibition of eNOS, it is generally accepted that high glucose induces a reduction in NO production and bioavailability and hence contributes to endothelial dysfunction.

Micro- and macrovascular beds are altered in diabetes by various changes in angiogenic mechanisms (Simons, 2005). However, from a vascular standpoint, diabetes is a paradoxical disease (Costa and Soares, 2013). Excessive angiogenesis contributes to the onset of both diabetic retinopathy (Wilkinson-Berka, 2004) and nephropathy (Osterby and Nyberg, 1987). Insufficient angiogenesis, however, plays a critical role in several pathological states including impaired wound healing process, weak neovascularization of coronary collaterals, embryonic vasculopathy during gestational diabetes, and acute transplant rejection in recipients suffering from diabetes (Galiano et al., 2004). Furthermore, diabetic neuropathy is a complication linked with reduced nutritive blood flow secondary to diabetes. Altered arteriogenesis, which is referred to as the process of remodeling (such as increase in diameter) of arteries, has also been widely observed in diabetics (Abaci et al., 1999). The weak mobilization of endothelial progenitor cells (EPC) from bone marrow and their defective function are also key features of diabetes that can cause aberrant neovascularization and hence contribute to the increase of cardiovascular risk (Tepper et al., 2002).

Angiogenesis is a process of formation of new capillary networks in response to reduced oxygen concentration (hypoxia) or pro-angiogenic molecular signals. This mechanism implicates the release of pro-angiogenic factors from both hypoxic endothelial cells and the their surrounding pericytes, which stimulate endothelial cell proliferation and sprouting of new vessels from existing ones (for review see Stapor et al., 2014). This is different from arteriogenesis, a mechanism involving the growth and development of existing arterioles following an acute vascular occlusion (Folkman and Shing, 1992). There are several equally important hypotheses for the mechanisms underpinning disturbances in angiogenesis in diabetes, including a downregulation of VEGF-A signaling response, alterations in inflammatory signals, activation of ER stress response, and accumulation of AGEs initiating oxidative stress and over-production of ROS (Tamarat et al., 2003). Accumulating data indicate that the impact of ROS on the vasculature depends essentially on the oxidative level of the environment. However, the precise amount of ROS needed to cause vascular dysfunction is difficult to predict. Under basal conditions, low ROS levels, especially hydrogen peroxide, can work as intracellular secondary messengers to modulate the action of pro-angiogenic factors such as VEGF-A; however, higher levels of ROS can conversely disturb neovascularization process (Griendling and FitzGerald, 2003a,b).

**FIGURE 8:**
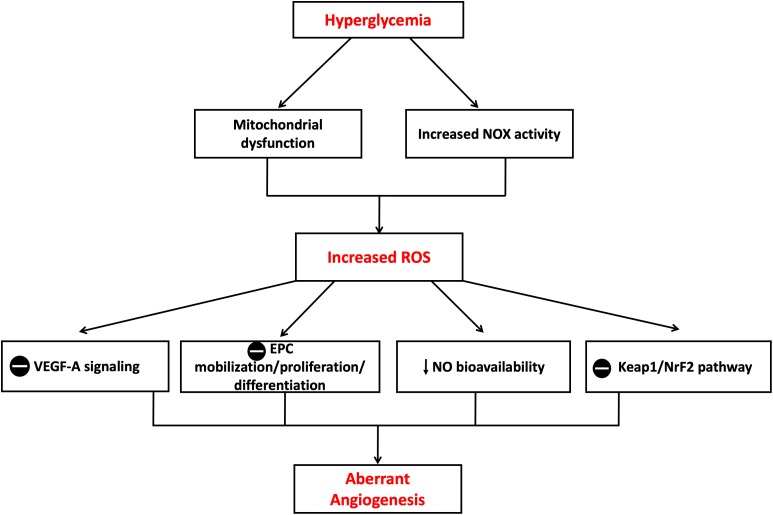
Mechanisms of aberrant angiogenesis induced in hyperglycemia. Increased ROS production in hyperglycemic environment both *in vitro* and *in vivo* is believed to cause insult on the angiogenic process. Theories proposed include suppression of VEGF-A signaling, suppression of EPC mobilization, proliferation and differentiation, reduction in NO bioavailability and suppression of Keap1/NrF-2 pathway with resultant suppression in HO-1 expression. EPC, Endothelial progenitor cell; Keap1, Kelch-like ECH-associated protein 1; NrF-2, nuclear factor erythroid 2–related factor 2; VEGF-A, Vascular endothelial growth factor A. The symbol 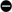 means inhibition.

Several ROS species, including O_2_^-^, H_2_O_2_, OH^-^ radicals, lipid peroxides, and ONOO^-^, have been recognized to play major roles in vascular biology controlling the angiogenic process (Tojo et al., 2005). Ebrahimian et al. (2006) reported that diabetes-mediated oxidative stress impaired post-ischemic neovascularization. Authors found that the reduction of ROS production restored key pro-angiogenic signaling pathways (e.g., VEGF-A). Nishikawa et al. (2000) proposed that high glucose-mediated mitochondrial production of ROS can stimulate several molecular mechanisms involved in aberrant angiogenesis. Other studies indicated that O_2_^-^ release by NOX complex plays a key role in VEGF expression and angiogenesis in a mouse model for diabetic ischemic vascular disease. In addition, increased expression of NADPH subunit NOX2 (gp91^phox^) has been found to correlate with increases in ROS and decreased VEGF-A in ischemic diabetic retinopathy (Al-Shabrawey et al., 2008). Furthermore, high levels of O_2_^-^ production by NOX are likely to scavenge NO or cause eNOS uncoupling and hence reduce NO bioavailability (Guzik et al., 2002). These changes in NO signaling pathway may also contribute to the regulation of post-ischemic angiogenic process because of the well-documented role of NO as a key modulator of bone marrow mononuclear cell (MNC) mobilization, differentiation and homing (Aicher et al., 2003). Tepper et al. (2002) reported that the phosphorylation and activation of p38 MAPK by diabetes-induced ROS production leads to impaired differentiation of bone marrow MNC into EPC *in vitro* and impaired their pro-angiogenic capacity *in vivo*. Diabetes has also been shown to activate p38 MAPK in vascular wall via signaling pathways that may involve PKC (Igarashi et al., 1999). The activation of p38 MAPK is known to downregulate EPC proliferation and differentiation which may further contribute to impaired angiogenesis in diabetes. Not only has development of oxidative stress with increased production of ROS been reported in *in vitro* and *in vivo* diabetic models of impaired angiogenesis, but also the anti-oxidant defense capacity is reduced which can also contribute to diabetes-induced oxidative stress (Wohaieb and Godin, 1987). Previous reports showed that chronic exposure of HUVECs to high glucose caused suppression of kelch-like ECH-associated protein (Keap)-1/erythroid 2–related factor (NrF)-2 pathway which is the major modulator of cytoprotective responses to stresses mediated by ROS including the HO system. The suppression of Keap1/NrF-2 correlated with impaired migration and tube-like formation capacity of HUVECs (Chen et al., 2015) (Figure 8).

## HO-1 and Its Protective Effects Against Diabetes-Mediated Endothelial Disturbances

Heme oxygenases are a group of anti-oxidant enzymes that play a pivotal role controlling intracellular levels of heme by mediating the biodegradation of heme to release the following byproducts, Fe^2+^, CO, and biliverdin in mammalian cells (Kikuchi et al., 2005; Ryter et al., 2006; Abraham and Kappas, 2008; Rochette et al., 2018). Biliverdin, a water-soluble molecule, is then metabolized by biliverdin reductase into bilirubin. In diabetes and other cardiovascular disorders such as hypertension, the excess of free heme leads to the formation ROS species, which in turn contribute to the onset of endothelial dysfunction. The HO system, by metabolizing the excess heme, can generate various byproducts, which may initiate different cytoprotective cardiovascular effects: (I) Pro-survival effect on endothelial cells; (II) Reduced inflammation and oxidative stress in vasculature; (III) Improved vascular homeostasis and control of the vascular tone regulation; and (IV) Enhanced neovascularization. Bilirubin and biliverdin are the two main active byproducts of HO action and are considered as the strongest scavengers of ROS in the body; however, CO can have anti-apoptotic and anti-inflammatory roles via the activation of guanylyl cyclase (Stocker et al., 1987; Nitti et al., 2017). CO is capable of suppressing the release of key inflammatory molecules such as of TNF-α and IL-1β and stimulating the synthesis of the human cytokine-synthesis inhibitory factor (CSIF or IL-10) (Otterbein et al., 2000). The last byproduct of heme degradation is free iron, which despite participating in Fenton reaction, which generates the very reactive OH^-^ radical, also activates Fe/ATPase pump, which pumps free iron outside of the cell and enhances protein expression of ferritin which can then scavenge free iron and exert therefore beneficial effects (Crichton et al., 2002).

Three HO isoforms have been reported in humans: HO-1 (32 KDa), which is the inducible form, and two other isoforms with constitutive expression [HO-2 (36 KDa) and a putative HO-3 (33 KDa)] (Nitti et al., 2017). The inducible HO-1, is expressed at very small amounts in normal tissues, except the liver and spleen, where it plays a key role in the elimination process of damaged and aged red blood cells (Gottlieb et al., 2012). Protein expression of HO-1 can be enhanced by many stimuli, which can induce ROS production. Among the pharmacological and chemical stimuli known to induce HO-1, there is heme itself [a byproduct of HO-1 action), heavy metals (e.g., cobalt], inflammatory cytokines, ultraviolet radiation, bacterial membrane component lipopolysaccharide (LPS), hyperglycemia, H_2_O_2_, cell growth factors, NO, and CO (Ryter et al., 2006). On the other hand, the expression of HO-2 is not induced by the factors inducing HO-1. However, HO-2 is constitutively expressed in high levels in certain tissues such as the brain, testis, smooth muscle cells and endothelial cells (Chen et al., 2003). In addition to maintaining heme homeostasis, basal amounts of HO-2 also play a key role in anti-oxidant cellular responses by regulating the activation of extracellular SOD, Akt, and ASK-1, through the modulation of heme degradation rate into its biological byproducts. Furthermore, HO-2 plays a pivotal role in sensing levels of oxygen and in cellular response to hypoxia (Munoz-Sanchez and Chanez-Cardenas, 2014). The protective role of HO-2 in hypoxia was demonstrated in endothelial cells. HUVECs and aortic endothelial cells exposed to hypoxia were found to exhibit a stable HO-2 protein expression despite a significant decline in its mRNA expression and a decrease in general protein translation. An increased interaction of HO-2 transcript with polysomes was observed during hypoxic events, leading to a selective activation of HO-2 protein translation, which maintained protein expression levels of HO-2. The maintenance of steady levels of HO-2 protected endothelial cells from apoptosis during hypoxic episodes (He et al., 2010). Finally, HO-3 is encoded by a pseudogene derived from the transcript of HO-2, but so far has only been identified in rats (McCoubrey et al., 1997).

The modulation of HO-1 expression involves a complex molecular interplay between different regulatory mechanisms. MAPK, phosphatidyl inositol 3-kinase (PI-3K)/Akt, protein kinases (PKA, PKC, and PKG) (Stocker and Perrella, 2006), NrF-2, activator protein (AP)-1, NF-κB, cyclic adenosine monophosphate-response element-binding (CREB) protein, and activation transcription factor (ATF)-2 are signaling pathways that have been found to play a role in gene regulation of HO-1 expression (Kim H.P. et al., 2011; Kim Y.M. et al., 2011). Of particular interest, in response to oxidative damage, NrF-2 controls the transcriptional induction of HO-1. NrF-2 is maintained in inactive form in the cytosol by interacting with protein Keap1, which prevents the nuclear translocation of NrF-2 and enhances proteasome-mediated degradation of NrF-2. Following induction of an oxidative stress state, NrF-2 is activated after its dissociation from Keap1; active NrF-2 translocates then to the nucleus and triggers the transcription of HO-1 gene. In support of the role of NrF-2 in controlling HO-1 expression, previous reports have found that fibroblasts from mice deficient in NrF-2 express low levels of HO-1 (Leung et al., 2003; Liu et al., 2007).

Several heavy metal protoporphyrins induce the expression of HO-1; however, Cobalt protoporphyrin (CoPP) is regarded as probably the most potent. CoPP indirectly modulates the production of two factors involved in regulating the expression of HO-1, NrF-2 and transcription regulator protein Bach1. Bach1 is a basic leucine zipper transcription factor that forms heterodimers with proteins from the Maf family and repress the transcription of the HO-1 gene. CoPP enhances the degradation of Bach1 protein. NrF-2 is also a member of the family of basic leucine zipper transcription factors, which is involved in protection against oxidative stress through its antioxidant response element (ARE)-directed induction of HO-1. CoPP also induces the overexpression of NrF-2 by decreasing its degradation, and being a basic leucine zipper transcription factor, the NrF-2 dimerizes with the Maf family transcription factors and together these heterodimers bind to ARE of the HO-1 promotor region resulting in enhancement in expression (Shan et al., 2006). Liu et al. (2013) also reported that CoPP increases the expression of the transcription factor forkhead box protein O1 (FOXO1), which results in transcriptional upregulation of HO-1 gene (Liu et al., 2013).

Besides this mRNA transcriptional activation of HO-1, it was found that HO-1 is also subjected to a post-transcriptional regulatory process which aims at reducing the expression of HO-1 to prevent that its overexpression becomes constitutive. This negative regulation of HO-1 expression is under the control of two microRNA (miR) molecules, miR-217 and miR-377, which work together to reduce the mRNA levels of HO-1 and hence reduce its protein expression. Knocking down these two microRNA molecules abrogated the attenuation of HO-1 expression (Beckman et al., 2011). Lin P.H. et al. (2008) showed that HO-1 expression is also regulated by a post-translational mechanism under the action of the ubiquitin/proteasome system. These findings were further supported by the increased HO-1 expression in the presence of proteasome inhibitor, MG-132, in a model of heme-induced oxidative damage (Chen and Regan, 2005).

### Role of HO-1 in Vascular Inflammation

It has been established that HO-1 is able to suppress inflammatory responses by the concomitant generation of CO and bilirubin, two anti-inflammatory molecules, and the removal of pro-inflammatory agent, heme. CO and bilirubin are reported to blunt both innate and adaptive immune responses by modulating the activation and functions of multiple cell types involved in inflammation such as immune cells, endothelial cells and platelets, which ultimately results in reduced recruitment and tissue infiltration of immune cells (Durante, 2011). The anti-inflammatory role of HO-1 was determined in HO-1 knockout mice. In these mice, HO-1 deficiency led to higher release of pro-inflammatory molecules (Kapturczak et al., 2004). In patients undergoing a surgery, higher levels of HO-1 were associated with lower levels of pro-inflammatory cytokine IL-6 (Taha et al., 2010). HO-1 was found to lower leukocyte rolling, adhesion, and transmigration to the sub-intimal space, by blunting the expression and function of surface adhesion molecules. In contrast, the inhibition of HO-1 caused the overexpression and over activation of adhesion molecules, activating thus leukocyte recruitment (Belcher et al., 2006). Multiple preclinical and clinical studies have been performed in many inflammatory vascular diseases to understand the impact of HO-1 overexpression on the pathogenesis and progression of the inflammatory process. Much evidence shows that the induction of HO-1 in the vessel wall inhibited the transmigration of monocytes to the sub-intimal space facilitated by oxidized low-density lipoproteins (LDL), leading to reduced atherosclerotic lesion buildup in mice lacking LDL receptor, a widely used model for atherosclerosis (Ishikawa et al., 2001). Circulating amounts of bilirubin in the general public were found to negatively correlate with the incidence of ischemic accidents (Mayer, 2000). This is not surprising knowing that bilirubin was found to decrease endothelial activation and dysfunction (Kawamura et al., 2005).

### Role of HO-1 in Angiogenesis

HO-1 and its gas byproduct CO have been recognized for their strong pro-angiogenic roles. Several pro-angiogenic molecules, including VEGF, were found to mediate their effects via the induction of HO-1. However, depending on the situation, the pro-angiogenic properties of HO-1 could be considered as beneficial or deleterious. For instance, favoring angiogenesis is critical for many physiological responses such as wound healing and neovascularization following an ischemic episode. However, activation of angiogenesis driven by HO-1 may be deleterious in other conditions such neoplastic states (Dulak et al., 2008).

In investigating the role of HO-1 in promoting angiogenesis, Zhao et al. (2011) found that partial deficiency of maternal HO-1 caused defects in the feto-maternal interface, structural defects in placental vasculature and deficiency in spiral artery remodeling. These alterations were not related to the fetal genetic background and were only dependent on the maternal genetic status of HO-1. Moreover, restoring blood flow and the reestablishment of optimal oxygen concentrations to an injury site are also achieved by a proper angiogenic process. The wound-healing sequence of events consists of multiple steps that all require an intact angiogenesis process (Bauer et al., 2005). Growth factors (e.g., VEGF), chemokines, and hypoxia-inducible factors (HIF) all contribute to the orchestration of the multi-step wound healing procedure (Tepper et al., 2005). The deletion of HO-1 in mice led to an impaired wound healing process compared to wild-type littermates. This was partly because of an impaired EPC mobilization capacity linked to capillary formation at the site of injury (Deshane et al., 2007). Furthermore, it has been noted that the natural activation of HO-1 expression in wounded skin areas was weaker and slower in diabetic mice compared to controls. In these animal models of diabetes, the local adenoviral-mediated delivery of HO-1, improved the wound healing process (Grochot-Przeczek et al., 2009). Furthermore, HO-1 was found to confer a cytoprotective role in ischemic cardiac tissue through the activation of protein expression of pro-angiogenic factors in infarcted areas of cardiac tissue (Zeng et al., 2010). Lin H.H. et al. (2008) observed that the transfer of the HO-1 gene to infarcted heart tissue confers protection, at least partly, through the activation of VEGF-A expression and the subsequent activation of angiogenesis. In addition, the overexpression of HO-1 in mesenchymal stem cells implanted in infarcted hearts, markedly reduced apoptosis and fibrosis in cardiac tissue and improved cardiac function and remodeling (Jeon et al., 2007). Recently, Maamoun et al. (2017) have reported that the incubation of HUVECs in intermittent high glucose conditions for 5 consecutive days caused the activation of ER stress response and an impaired capacity of cells to form tube-like structures on a 3-dimensional matrigel matrix. This effect was associated with a decrease in the mRNA and protein expression of VEGF-A, indicating reduced angiogenesis capacity in these cells. Authors found that the pharmacological induction of HO-1 with CoPP reduced ER stress response and prevented the effects of high glucose-mediated ER stress on angiogenic capacity of cells, further stressing the pro-angiogenic effects of HO-1 in endothelial cells (Maamoun et al., 2017).

It is crucial to also highlight that despite the evidence in literature in support of the beneficial role of HO-1 in angiogenesis control, some reports identified a negative impact of HO-1 in tumoral neovascularization. Some human tumors, such as renal cell carcinoma and prostate cancer, exhibit elevated expression levels of HO-1 (Goodman et al., 1997). HO-1 could therefore contribute to tumor cell growth, survival and progression and ultimately participate in the resistance to drug therapy (Nowis et al., 2006). In these conditions, the inhibition of HO-1 reduced tumor development in murine cancer models indicating a potential therapeutic role for HO-1 inhibition in certain cancer types (Dulak et al., 2008). However, there are also some reports that show HO-1 may play an anti-angiogenic role in certain cancer situations. In prostatic cancer cells (PC3), Ferrando et al. (2011) found that the overexpression of HO-1 caused the downregulation of several pro-inflammatory and angiogenic factors (NF-κB, VEGF-A, VEGF-C). To model the *in vivo* angiogenic process, the authors intradermally inoculated PC3 cells, that had been transfected with a stable HO-1 DNA construct, into immunodeficient mice and found that these cells produced tumors that were less richly vascularized than controls (Ferrando et al., 2011). However, in other conditions driven by excessive angiogenesis such as diabetic retinopathy (Wilkinson-Berka, 2004) and nephropathy (Osterby and Nyberg, 1987), HO-1 induction was still reported to be protective, indicating the complexity of the physiological actions of HO-1. For instance, Fan et al. (2012) observed in streptozotocin-injected diabetic rats pre-injected with hemin, a HO-1 inducer, that retinal ganglion cells were less prone to apoptosis compared to controls (not pre-treated with hemin), suggesting that HO-1 induction in rat retinas protected against diabetes-mediated neurodegeneration. Furthermore, the expression levels of VEGF, a strong pro-angiogenic factor which contributes to exaggerated angiogenesis, was also attenuated in retinas from streptozotocin-injected rats treated with hemin (Fan et al., 2012).

Taken together, data outlined here identify HO-1 as a key modulator of the angiogenic process in different pathophysiological conditions. Therefore, HO-1 induction could be a potential therapeutic target for the inhibition of angiogenesis in carcinogenic conditions, as opposed to being a therapeutic target for the induction of angiogenesis in diabetic cardiovascular complications. These studies also highlight the complexity of signaling pathways underpinning the action of HO-1 in angiogenesis.

### Role of HO-1 in Oxidative Stress

The most well-known properties of HO-1 are its anti-oxidant functions (Hayashi et al., 1999). Of particular note, the work conducted by Ishikawa et al. (2012) demonstrated that mice lacking the gene of HO exhibited a weaker anti-oxidant capacity and an extensive lipid peroxidation. The anti-oxidant capacity of HO-1 is due to its byproducts biliverdin and CO. Bilirubin strongly scavenges several oxygen free radicals including singlet oxygen, O_2_^-^, ONOO^-^ and RO_2_ radicals. Moreover, unlike GSH, bilirubin, because of its lipophilic nature, is closely associated with cell membranes, and hence protects them against lipid damage by peroxidation. Bilirubin was found to work better as an O_2_^-^ scavenger and inhibitor of ONOO^-^-mediated protein nitration than biliverdin (Jansen et al., 2010). In addition to its ROS scavenging properties, some studies proposed that the protective actions of bilirubin are also partly related to its capacity to inhibit inducible NOS (iNOS), which eventually leads to less production of the highly reactive and potent ONOO^-^ free radical (Wang et al., 2004). CO also mediates part of the global anti-oxidant actions of HO-1 (Srisook et al., 2006). CO was reported to block ONOO^-^-mediated protein nitration in endothelial cells through heightened intracellular GSH levels (Li et al., 2007). The anti-oxidant role of HO-1 was further demonstrated recently in HUVECs treated with intermittent high glucose. Authors reported that the induction of HO-1 using CoPP prevented high glucose-mediated concomitant increase in superoxide production and reduction in NO release, indicating an improved NO bioavailability. These focal effects were associated with a decrease in ER stress response activation, reduced phosphorylation of NOX regulatory subunit p47^phox^ at Ser345, and an enhanced phosphorylation of eNOS at its activatory site (Ser1177) (Maamoun et al., 2017).

There is accumulating evidence that oxidative stress contributes to the early stages as well as the maintenance and progression of diabetic cardiovascular complications. Therefore, HO-1, owing to its strong anti-oxidant properties, may be utilized as a viable and promising therapeutic target in these conditions. The protective actions of HO-1 induction have been demonstrated in animal models of diabetic cardiomyopathy and vasculopathy (Li et al., 2012). Moreover, *in vitro* studies have demonstrated the highly potent anti-oxidant effect of bilirubin with increased protection of vascular endothelial cells, where even concentrations of bilirubin in the nanomolar range protected cells against oxidative stress damage both through direct scavenging of ROS radicals and indirectly by blocking the activity of NOX complex (Abraham and Kappas, 2008).

### Role of HO-1 in Apoptosis

A high percentage of apoptotic endothelial cells was observed in animal models of diabetes-mediated vascular injury (Negi et al., 2011). Clinical data also suggest the involvement of apoptosis in diabetic vascular diseases where the expression of pro-apoptotic molecules was high while in contrast the expression of anti-apoptotic ones was low (Arya et al., 2011). HO-1 mediates its anti-apoptotic action through multiple molecular mechanisms including the inhibition of oxidative stress, ER stress and inflammation. NF-κB was shown to control cellular apoptosis in addition to its well-known role in inflammation. HO-1 induction by CoPP was found to reduce the expression of IL-6, a gene regulated by NF-κB, and CHOP in response to stressful stimuli such as ischemia-reperfusion injury, with the consequent inhibition of apoptosis (Ben-Ari et al., 2013). Other mechanisms suggested for the anti-apoptotic effects of HO-1 include blunting the apoptotic signaling pathway mediated by the interaction of TNF-α with its receptor (Jaeschke and Woolbright, 2013). These anti-apoptotic actions of HO-1 may be driven by the release of CO, which was shown to protect against apoptosis in many cell types, including endothelial cells, fibroblasts, hepatocytes and pancreatic β-cells. Brouard et al. (2000) were able to show that HO-1 inhibits apoptosis, namely by generating CO. The inhibition of HO-1 activity by tin protoporphyrin (SnPPIX) failed to prevent endothelial cell apoptosis. The direct effect of CO on apoptosis was confirmed by showing that exposing endothelial cells to exogenous CO, while inhibiting HO-1 activity by SnPPIX, prevented apoptosis. Moreover, investigators have also explored some of the signaling pathways behind the action of HO-1/CO, and showed that it is partially mediated by the activation of p38 MAPK signaling transduction pathway. This was further supported by the observation that the selective blockade of p38 MAPK by the over-expression of a dominant negative mutant resulted in the abolishment of the anti-apoptotic effect of HO-1 (Brouard et al., 2000). In addition, as previously discussed, in diabetic vascular disease, oxidative stress occurs in endothelial cells which subsequently leads to apoptosis. Hence, HO-1 by merely exerting its anti-oxidant effects is reported to protect these cells from apoptosis (Schmeichel et al., 2003). Other studies focused on the role of bilirubin in this pro-survival behavior of HO-1. It has been shown that bilirubin was found to mediate its anti-apoptotic role by upregulating extracellular-signal-regulated kinase (ERK) MAPK and PI-3K/Akt/eNOS pathways (Hung et al., 2010).

More recently, pharmacological induction of HO-1 using CoPP was reported to protect endothelial cells from high glucose-mediated apoptosis through the alleviation of ER stress response. Maamoun et al. (2017) observed that the exposure of HUVECs to intermittent high glucose for a duration of 5 days led to cell death, while induction of HO-1 prevented this deleterious manifestation. The cytoprotective effects of HO-1 were associated with a reduction in the activation of PARP-1 and caspases 3 and 7. Furthermore, HO-1 induction in HUVECs prevented the activation of NF-κB and JNK pathways caused by high glucose treatment, indicating reduced cellular inflammation in the presence of HO-1 (Maamoun et al., 2017). While the amount of scientific evidence is still relatively small, some studies suggest HO-1 activation as a novel and promising strategy to improve survival of endothelial cells through HO-1 byproducts, bilirubin and CO, which may delay the development of various diabetic cardiovascular complications.

## Conclusion

Crosstalk between oxidative stress and ER stress significantly contributes to the onset and development of endothelial dysfunction and altered angiogenesis and therefore represent major therapeutic targets for alleviating micro- and macrovascular complications associated with this metabolic disturbance. HO-1 carries out, anti-oxidant, anti-inflammatory, anti-apoptotic and angiogenic actions, through its by-products CO and bilirubin, and can thus affect therefore multiple cellular pathways involved in endothelial dysfunction, including oxidative stress and ER stress response. As a multi-target molecule affecting major aspects of cardiovascular dysfunction, HO-1 induction represents a potential therapeutic approach for cardiovascular complications associated with diabetes.

## Author Contributions

HM and AA wrote the manuscript and constructed the figures. TB, GP, and SM contributed to selected sections and critically revised the manuscript. All authors approved the final version for submission.

## Conflict of Interest Statement

The authors declare that the research was conducted in the absence of any commercial or financial relationships that could be construed as a potential conflict of interest.
